# “One-Stop” Simultaneous TEVAR and Renal Denervation for Stanford Type B Aortic Dissection Complicated by Refractory Hypertension

**DOI:** 10.1016/j.jaccas.2026.108981

**Published:** 2026-06-25

**Authors:** Lin Chen, Hongbin Lin, Xiaofang Chen, Mingchen Sun, Zhaokai Li, Xin Su, Hua Peng, Yan Wang, Ye Cheng

**Affiliations:** Department of Cardiology, The Xiamen Cardiovascular Hospital of Xiamen University, Fujian, China

**Keywords:** Nagamine classification, refractory hypertension, renal denervation, Stanford type B aortic dissection, thoracic endovascular aortic repair

## Abstract

**Background:**

The management of Stanford type B aortic dissection complicated by refractory hypertension remains clinically challenging.

**FIH Summary:**

A 56-year-old male with type B aortic dissection and refractory hypertension underwent a “one-stop” procedure combining thoracic endovascular aortic repair (TEVAR) and bilateral renal denervation (RDN). Despite complex anatomy involving the right renal artery (Nagamine III-a), RDN was successfully performed. At 3-month follow-up, blood pressure improved from 177/111 mm Hg to 129/83 mm Hg with stable aortic morphology.

**Discussion:**

RDN addresses sympathetic hyperactivity while TEVAR provides mechanical stabilization, effectively breaking the hypertension-dissection cycle. Precise anatomical assessment is critical for RDN feasibility in dissected renal arteries.

**Novelty:**

This report demonstrates the feasibility of a single-session “one-stop” TEVAR and RDN, even when the dissection involves the renal artery origins.

**Take-Home Message:**

Meticulous preoperative anatomical assessment using the Nagamine classification and multidisciplinary team collaboration is essential for ensuring procedural safety and efficacy.

## Visual Summary

**Figure:**
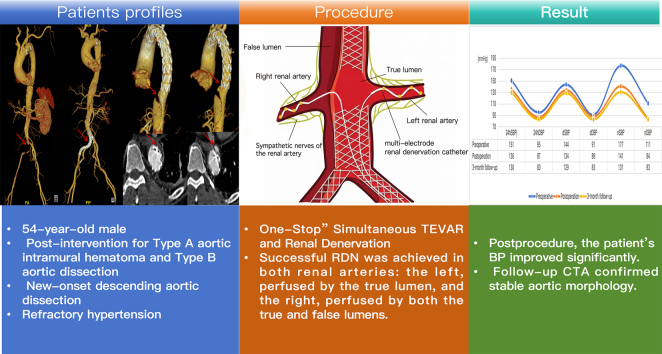

**Visual Summary:**
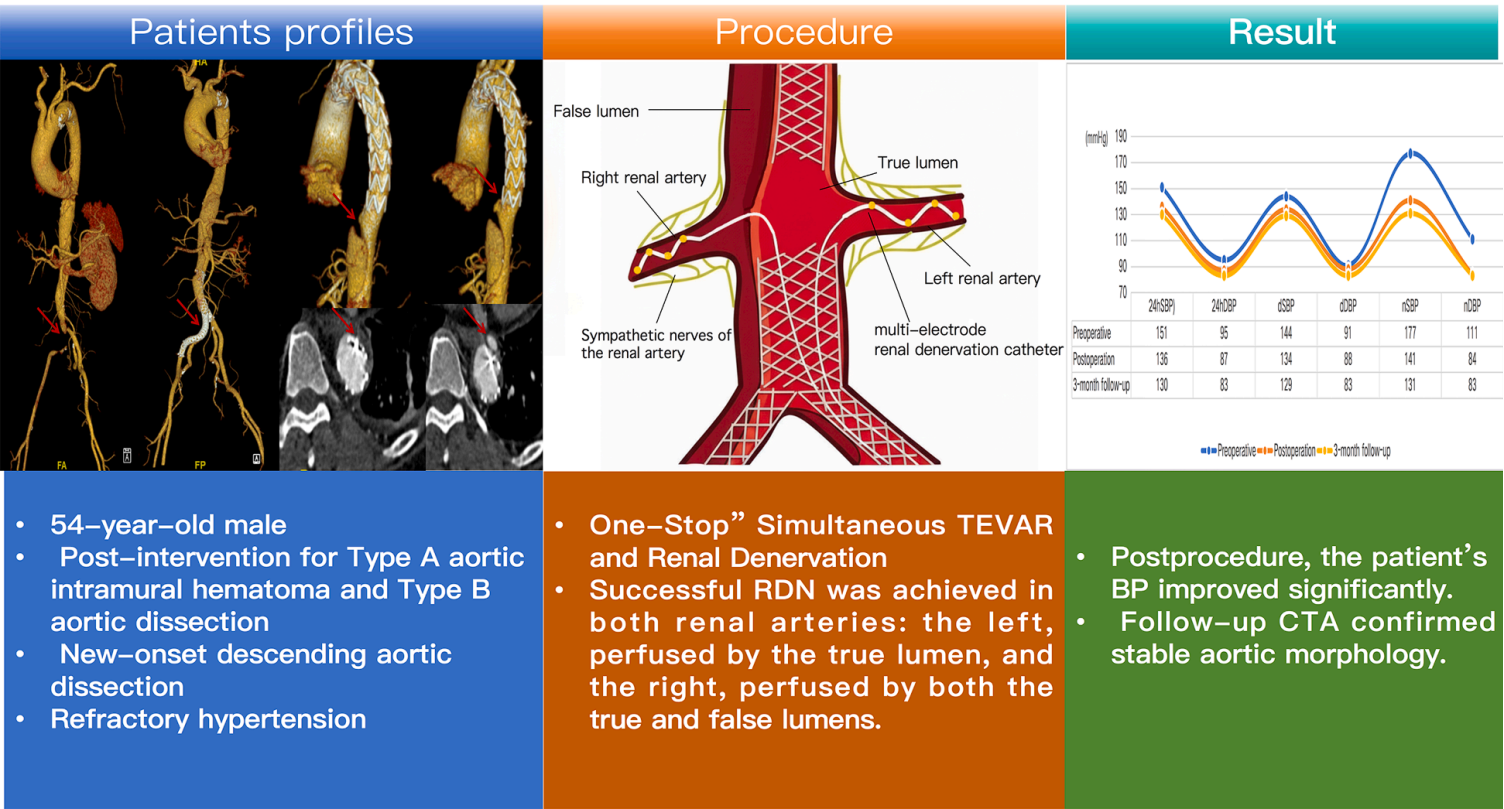
Clinical Summary of “One-Stop” Simultaneous TEVAR and RDN The panels outline the profile of a 54-year-old male with descending aortic dissection and refractory hypertension (left), the schematic of the simultaneous endovascular procedure (center), and the successful clinical outcomes, demonstrating significant and sustained blood pressure reduction alongside stable aortic morphology (right). BP = blood pressure; CTA = computed tomography angiography; RDN = renal denervation; TEVAR = thoracic endovascular aortic repair.

Renal denervation (RDN) is effective for refractory hypertension (RH), yet its role in patients with comorbid Stanford type B aortic dissection (TBAD) remains undetermined. Our team successfully performed a “one-stop” simultaneous thoracic endovascular aortic repair (TEVAR) and RDN in a TBAD patient with right renal artery involvement. Postoperatively, the patient achieved marked blood pressure (BP) reduction and stable aortic morphology, demonstrating the potential of this integrated strategy.

**Take-Home Messages**

• Simultaneous “one-stop” thoracic endovascular aortic repair and renal denervation is a promising strategy for type B aortic dissection complicated by refractory hypertension, as it effectively breaks the vicious cycle between aortic dissection and uncontrolled blood pressure.
• Meticulous preoperative anatomical assessment using the Nagamine classification and multidisciplinary team collaboration is essential to ensure procedural safety and efficacy.

## Case Summaries

A 56-year-old male with a >10-year history of poorly controlled hypertension presented to the emergency department with sudden-onset abdominal pain accompanied by right-lower-extremity pain for 1 day. Emergency computed tomography angiography (CTA) confirmed a Stanford type A aortic intramural hematoma combined with a TBAD. The true lumen of the right iliac artery was severely compressed and nearly occluded. Following admission, the patient underwent emergent endovascular stenting of the abdominal aorta and right iliac artery, followed by a TEVAR for aortic intramural hematoma 3 weeks later. The residual descending and abdominal aortic dissection was managed with conservative medical therapy.

**Figure 1:**
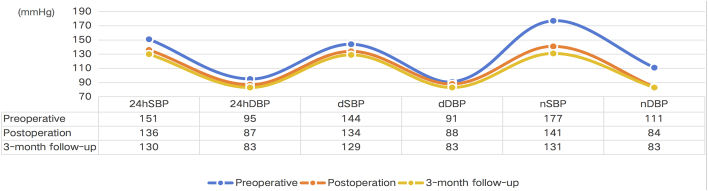
Results of 24-Hour Ambulatory Blood Pressure Monitoring Before Treatment, After Treatment, and at 3-Month Follow-Up 24hSBP = 24-hour average systolic pressure; 24 h DBP = 24-hour average diastolic pressure; dSBP = daytime average systolic pressure; dDBP = daytime average diastolic pressure; nSBP = nighttime average systolic pressure; nDBP = nighttime average diastolic pressure.

Comprehensive evaluation excluded common secondary causes of hypertension. Despite lifestyle modifications and a regimen of 4 antihypertensive medications (metoprolol succinate 47.5 mg/day, terazosin 2 mg/day, nifedipine controlled-release 60 mg/day, and sacubitril/valsartan 200 mg/day), the patient's BP remained poorly controlled. Ambulatory blood pressure monitoring (ABPM) revealed marked BP variability and an extreme reverse-dipping pattern, with an average daytime BP of 144/91 mm Hg and a paradoxically higher average nighttime BP of 177/111 mm Hg (Figure 1).

**Figure 2:**
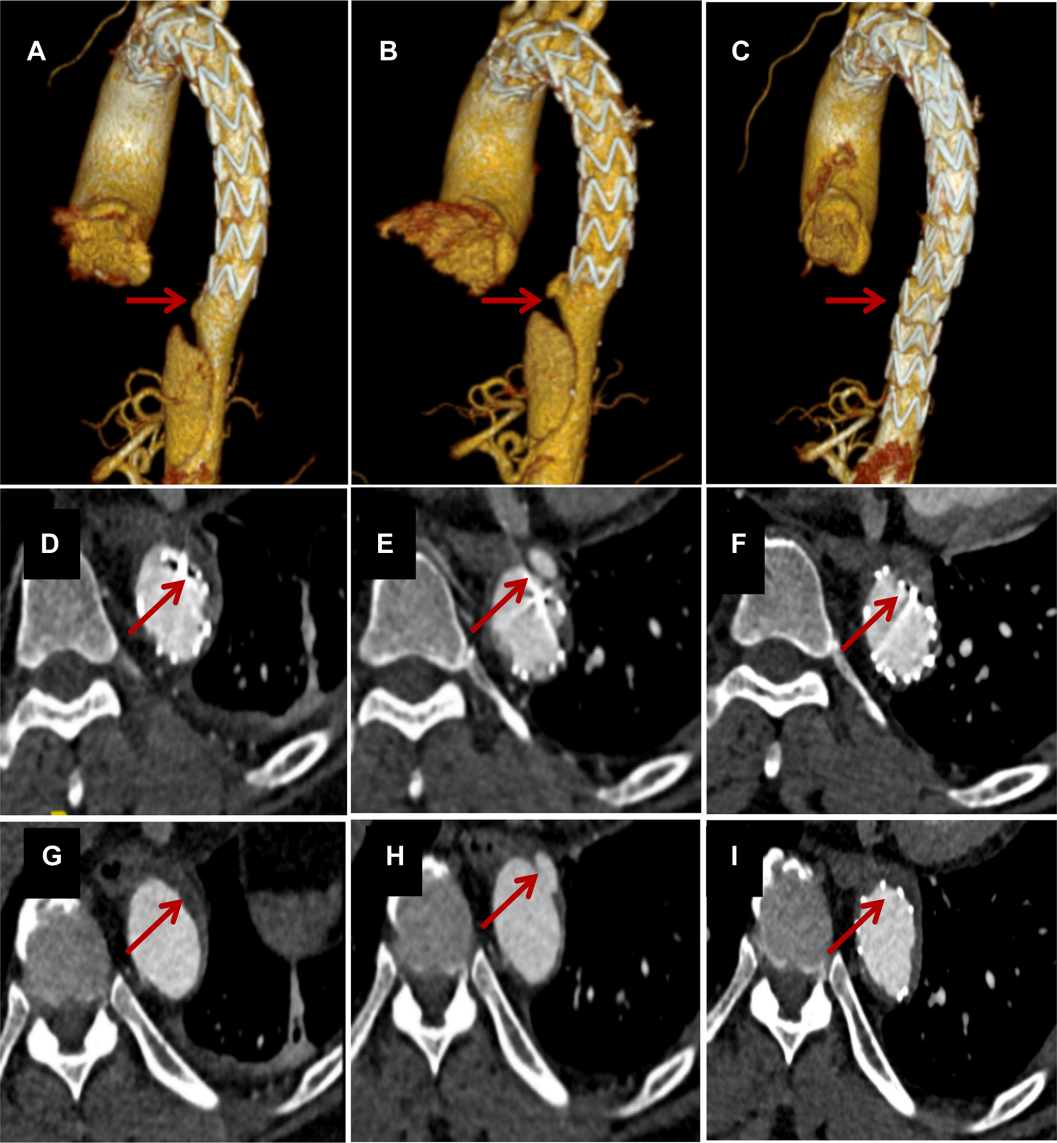
Longitudinal Computed Tomography Angiography Assessment of the Descending Aorta (A, D, G): Initial post-thoracic endovascular aortic repair imaging showing no distal dissection; (B, E, H): 1-month follow-up revealing a newly developed distal intimal tear below the prior stent graft, driving dissection progression.Red arrows show imaging changes before, after progression, and following surgery. (C, F, I): 3-month follow-up after the “one-stop” procedure, demonstrating complete resolution of the dissection and stable stent graft placement, validating the mechanical success of the secondary intervention.

One month post TEVAR, a follow-up thoracoabdominal CTA revealed a newly developed distal dissection just below the previous thoracic stent graft (Figure 2).

Following a multidisciplinary team (MDT) discussion, it was concluded that isolated endovascular repair was insufficient for lesion stabilization. RDN was deemed essential to achieve intensive BP control and disrupt the “vicious cycle” of dissection progression. To minimize risks of repeated anesthesia, puncture complications, and cumulative contrast exposure—and respecting the patient's preference against multiple surgeries—a “one-stop” simultaneous TEVAR and RDN procedure was planned, as the TEVAR risk was manageable and did not interfere with RDN execution.

Under general anesthesia, a distal TEVAR was initially performed via left femoral artery access to seal the new intimal tear and mechanically stabilize the dissection. Subsequently, utilizing the same access, RDN was executed using the Symplicity Spyral multielectrode RDN system (Medtronic).

**Figure 3:**
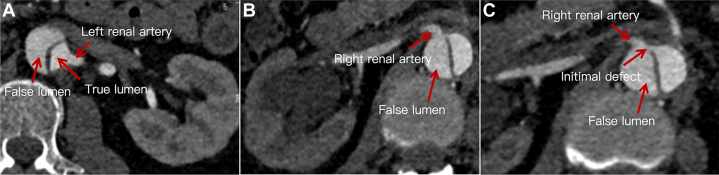
High-Resolution Computed Tomography Angiography Axial Views of Renal Artery Involvement (A) The left renal artery originates exclusively from the true lumen. (B and C) The right renal artery exhibits a complex origin; although its orifice is involved by the false lumen, an intimal defect (arrows) provides a patent perfusion channel from the true lumen. This anatomical identification (Nagamine subtype III-a) is the decisive factor for the feasibility of renal denervation access.

**Figure 4:**
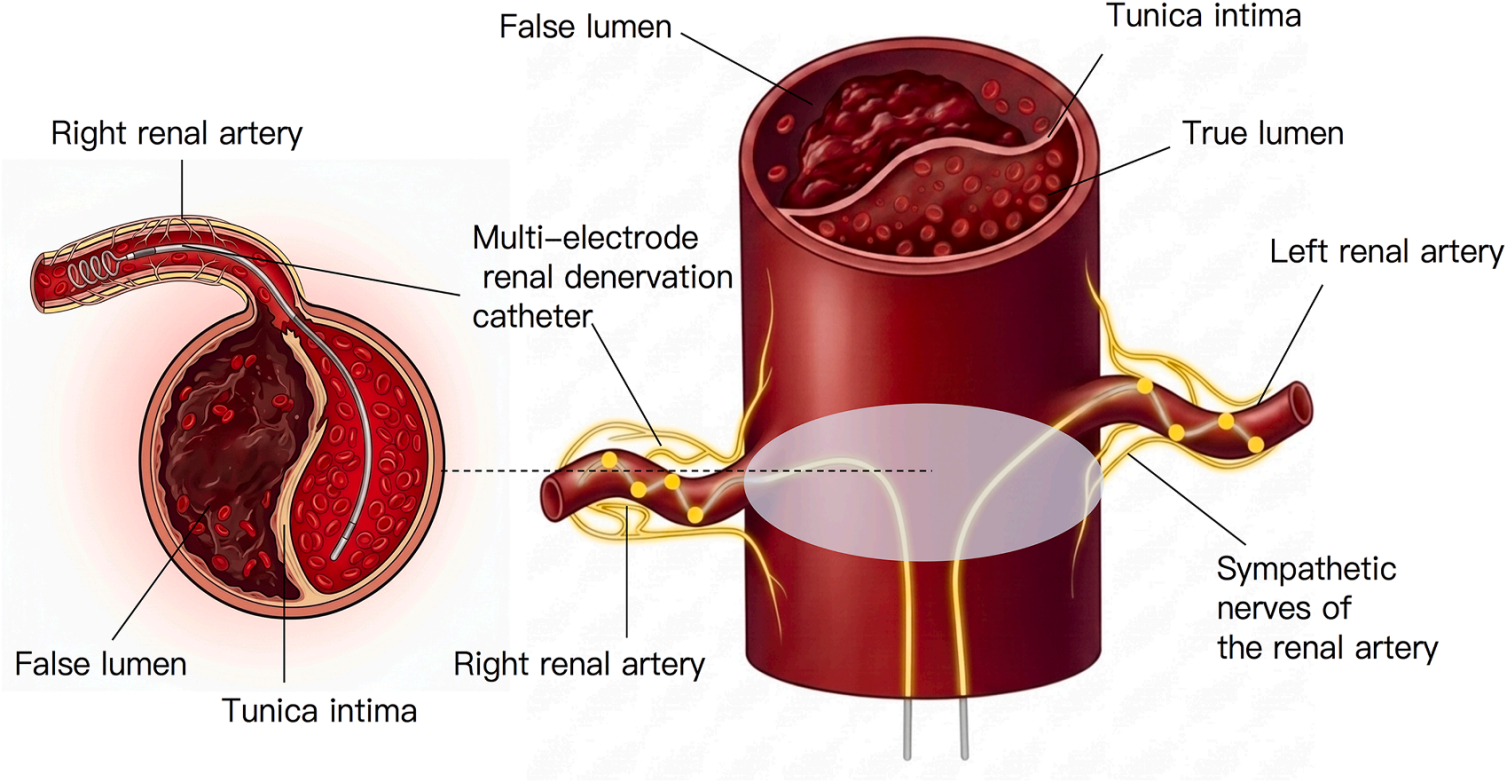
Schematic Representation of Renal Denervation The diagram illustrates the catheter path: access to the left renal artery is straightforward via the true lumen, while the right renal artery requires navigation through the intimal tear.

Preoperative CTA demonstrated that the left renal artery was supplied by the true lumen, whereas the right renal artery received mixed perfusion from both the true and false lumens (Figure 3). Crucially, a patent perfusion channel existed between the abdominal aortic true lumen and the right renal artery, and the entire course of the right renal artery exhibited no obvious structural abnormalities. These anatomical features made RDN of the right renal artery feasible (Figure 4).

**Figure 5:**
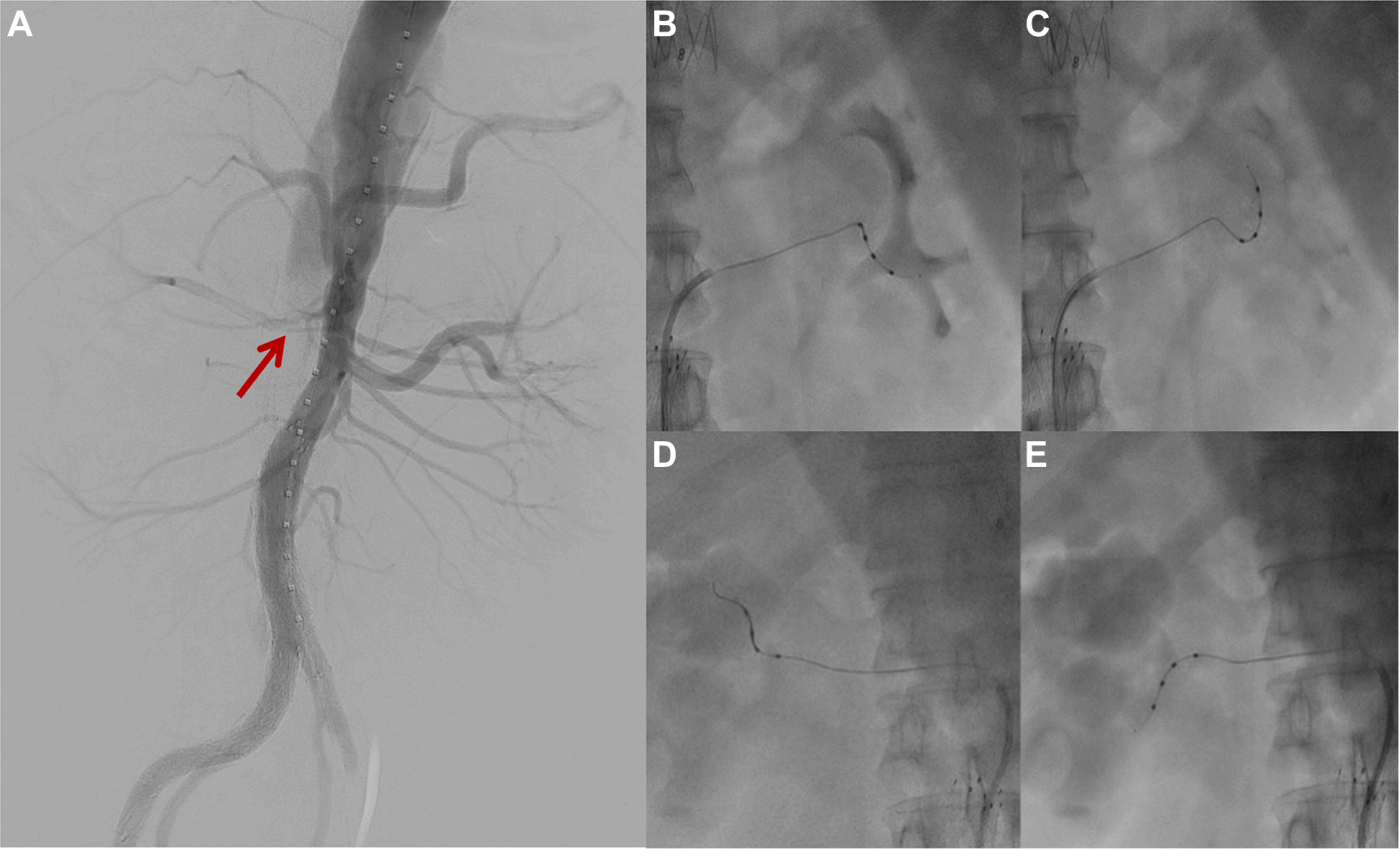
Procedural Snapshots of Bilateral Renal Denervation (A) No stenosis is observed in the left renal artery, while the right renal artery exhibits reduced perfusion compared to the left (arrow). (B-E) Step-by-step deployment of the multielectrode ablation catheter in the left and right renal arteries. Note the 5-mm safety margin maintained near the ostium of the right renal artery to prevent radiofrequency-induced damage to the fragile intimal flap.

A renal double curve guiding catheter was advanced to the ostium of the left renal artery. Supported by a THUNDER guidewire, the multielectrode ablation catheter was deployed to achieve comprehensive ablation of the left renal artery, with a total of 25 radiofrequency ablation sites performed. Although the right renal artery partially originated from the false lumen, the renal double curve guiding catheter was carefully navigated across the intimal tear to engage the right renal ostium. Assisted by a THUNDER guidewire, the multielectrode RDN catheter was deployed to achieve complete ablation of the right renal artery, with a total of 22 sites ablated. Intraoperatively, circumferential ablation was performed on all branches >3 mm in diameter. The total contrast volume used was 80 mL (Figure 5, Video 1).

Performing RDN in the right renal artery was technically challenging. To ensure procedural stability, a 7F guiding catheter was utilized. We strictly monitored the pressure waveform to confirm the catheter tip remained within the true lumen, while employing gentle contrast injections to minimize the risk of iatrogenic injury. Crucially, a 5-mm safety margin was maintained to avoid ablation near the false lumen. Emergency implantation of a covered renal stent was prepared as a bailout strategy in case of acute renal malperfusion; fortunately, this complication did not occur.

Immediate post-TEVAR angiography confirmed the complete sealing of the distal intimal tear. Following RDN, the patient's BP immediately dropped to 120/70 mm Hg. The postoperative antihypertensive regimen was adjusted to nifedipine controlled-release (30 mg/day) and metoprolol succinate (47.5 mg/day). Postoperative ABPM revealed an average daytime BP of 134/88 mm Hg and an average nighttime BP of 141/84 mm Hg.

At the 3-month follow-up, ABPM demonstrated an average daytime BP of 129/83 mm Hg and an average nighttime BP of 131/83 mm Hg (Figure 5). Follow-up CTA revealed no new aortic lesions and demonstrated that the morphology and perfusion of the renal arteries remained unchanged, with no evidence of stenosis, thrombosis, or intimal tear. Monthly BP follow-ups will be conducted, with a follow-up aortic CTA scheduled for 1 year postoperatively.

## Novelty of the Submission

This case demonstrates the successful management of a highly challenging patient with Stanford TBAD complicated by RH. Through an innovative “one-stop” approach combining TEVAR and RDN, we successfully sealed the new distal intimal tear driving dissection progression while safely and effectively controlling the life-threatening BP abnormalities.

A classic “vicious cycle” exists between the progression of aortic dissection and RH: The immense shear stress caused by hypertension continuously tears the aortic intima, leading to disease progression; conversely, the involvement of renal arteries by the dissection causes relative renal ischemia, which strongly activates the sympathetic nervous system and the renin-angiotensin-aldosterone system, leading to further BP elevation and severe fluctuations.^1^ The “one-stop” strategy employed in this case provided immediate mechanical stabilization via TEVAR, while simultaneously addressing sympathetic hyperactivity via RDN. This combined, single-session procedure achieved a dual, precise “anatomical and physiological” intervention for aortic disease.

**Figure 6:**
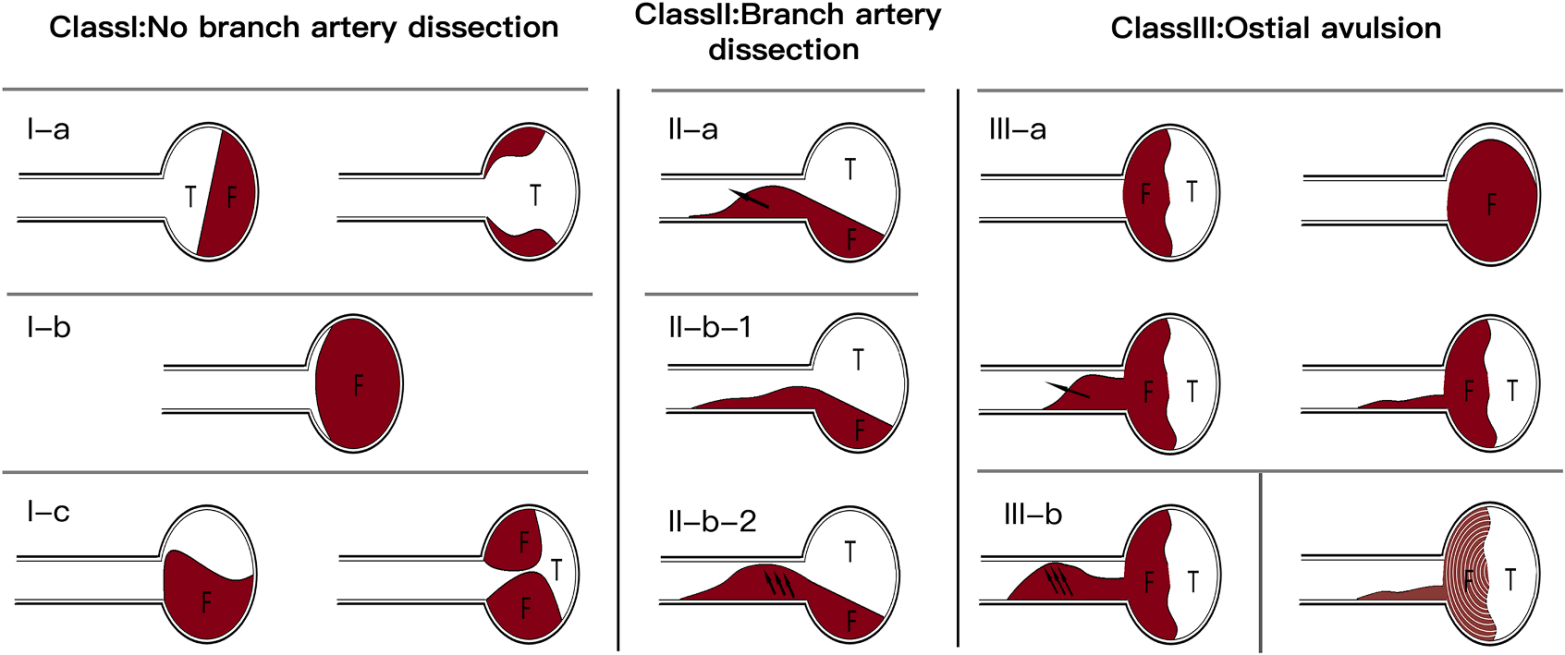
The Nagamine Classification System Used for Preoperative Risk Stratification Our case involved Subtype I-a and Subtype III-a, both characterized by the presence of an effective true-lumen perfusion channel. This classification is clinically essential for identifying suitable candidates and avoiding contraindications (e.g., Subtypes III-b/c) for renal denervation in aortic disease.

The most innovative and challenging technical highlight of this case was performing RDN on the right renal artery involving dissection. In 2012, Nagamine et al. proposed a classification system comprising 3 main types and 9 subtypes based on the morphological relationship between the dissection intimal flap and the branch arteries^2^ (Figure 6). Among these, subtypes I-a, II-a, II-b-1, and III-a retain an effective blood perfusion channel between the true lumen of the abdominal aorta and renal artery. In our case, the left renal artery was classified as subtype I-a, and the right as subtype III-a, both maintaining true lumen perfusion. It was precisely this specific anatomical foundation that allowed our catheter to access the true lumen of the renal artery for ablation. In contrast, subtypes I-b and I-c have significantly compressed perfusion channels, theoretically making RDN extremely difficult and risky, with no reports in the literature to date. Subtypes III-b and III-c, completely lacking effective perfusion channels, should be considered contraindications for RDN. Therefore, meticulous preoperative anatomical evaluation based on CTA is crucial.

The intimal flap in aortic dissection is extremely fragile, and radiofrequency energy could potentially cause intimal tearing, dissection rupture, or localized thrombosis.^3^ In this case, to maximize safety, we adopted a strict safety-margin strategy during the procedure: intentionally sparing a 5-mm area near the renal artery ostium adjacent to the false lumen to prevent radiofrequency energy from exacerbating the dissection at the abdominal aortic false lumen and the renal artery ostium. Postoperative and follow-up CTA revealed no dissection progression, and the renal arteries experienced no adverse events related to thermal injury (e.g., intimal tear, thrombosis, or luminal restenosis).

Literature reporting RDN in patients with aortic dissection remains sparse; however, available evidence consistently demonstrates significant BP reduction.^4^^,^^5^ In these previous reports, the target vessels predominantly focused on renal arteries with normal true-lumen perfusion.^4^ In contrast, this case successfully challenged a complex dissection anatomy. We conclude that the key factors for the successful implementation of bilateral RDN in this case are, first, the presence of an effective channel from the aortic true lumen into the renal artery true lumen, and second, the absence of severe structural damage to the main renal artery and its branches. Furthermore, in-depth preoperative discussions by MDT, particularly the precise guidance from the radiology department regarding the intimal tear and hemodynamics, provided decisive assistance in formulating the surgical strategy.

Future directions: Further large-scale, prospective studies are warranted to validate the long-term safety and efficacy of “one-stop” TEVAR and RDN in patients with complex aortic dissections.

Future research should focus on establishing standardized protocols and anatomical criteria for RDN in the setting of dissected renal arteries to ensure predictable clinical outcomes.

## Conclusions

In summary, the “one-stop” TEVAR combined with RDN is a highly promising therapeutic strategy for patients with aortic dissection complicated by RH and complex anatomy. Furthermore, this case underscores the critical role of preoperative MDT collaboration and meticulous imaging evaluation regarding the extent of renal artery involvement.

## Funding Support and Author Disclosures

This study was funded by the Natural Science Foundation of Xiamen Municipality (3502Z20227295) and Natural Science Foundation of Fujian Province (2025J011490). The authors have reported that they have no relationships relevant to the contents of this paper to disclose.

## Appendix

Video 1 Renal Denervation Procedure
